# Incidence of small-for-gestational-age newborns in pregnant women with COVID-19

**DOI:** 10.61622/rbgo/2025rbgo20

**Published:** 2025-04-30

**Authors:** Gustavo dos Santos Raupp, Renato Teixeira Souza, Maria Laura Costa, Jose Guilherme Cecatti, Annerose Barros, Ellen Machado Arlindo, Edson Vieira Cunha, Janete Vettorazzi

**Affiliations:** 1 Hospital Moinhos de Vento Porto Alegre RS Brazil Hospital Moinhos de Vento, Porto Alegre, RS, Brazil.; 2 Universidade Federal do Rio Grande do Sul Porto Alegre RS Brazil Universidade Federal do Rio Grande do Sul, Porto Alegre, RS, Brazil.; 3 Universidade Estadual de Campinas Department of Obstetrics and Gynecology Campinas SP Brazil Department of Obstetrics and Gynecology, Universidade Estadual de Campinas, Campinas, SP, Brazil.; 4 Hospital de Clínicas de Porto Alegre Porto Alegre RS Brazil Hospital de Clínicas de Porto Alegre, Porto Alegre, RS, Brazil.

**Keywords:** COVID-19, Coronavirus infections, Pregnancy complications, Maternal health, Infant, small for gestational age, Infant, newborn

## Abstract

**Objective:**

This study aimed to assess the incidence of small for gestational age (SGA) newborns in pregnant women infected with COVID-19 and examine the associated neonatal outcomes.

**Methods:**

This study involved a secondary analysis of the REBRACO Network, a prospective cohort study conducted in 15 maternity hospitals in Brazil before the introduction of COVID-19 vaccination (February 2020 to February 2021). Demographic data of pregnant women tested for COVID-19 were analyzed, and fetal outcomes were compared between women with positive and negative COVID-19 results who had SGA fetuses.

**Results:**

A total of 729 symptomatic pregnant women with COVID-19 were included in the study. However, there were 248 participants with missing information regarding childbirth or loss of follow-up, and 107 participants without confirmatory tests for COVID-19. Among the remaining participants, 198 had confirmed COVID-19 and 176 tested negative. The incidence of SGA among women with COVID-19 was 22.4%, whereas the incidence among women who tested negative for COVID-19 was 14.8%. SGA newborns born to COVID-19 positive pregnant women were 1.6 times more likely to experience adverse outcomes (such as prematurity, stillbirth, neonatal death, and admission to a neonatal ICU) compared to non-SGA newborns [OR = 1.655 (1.145 – 2.394); P=0.017]. In SGA newborns of pregnant women with confirmed COVID-19 infection, mechanical ventilation use was found to be associated with the infection [OR = 0.692 (0.562 – 0.853); P=0.002].

**Conclusion:**

The higher incidence of SGA newborns and its stronger association with prematurity in pregnant women with confirmed COVID-19 infection suggest that COVID-19 infection is a significant factor contributing to neonatal morbidity and mortality.

## Introduction

The COVID-19 pandemic had a significant impact on the global population's health. The disease manifests with a wide range of symptoms, from asymptomatic to severe illness, commonly including cough, fever, myalgia, sore throat, and headache. In severe cases, it can lead to pneumonia, septic shock, and multiple organ failure. Consequently, special attention has been given to specific populations by healthcare professionals, including pregnant women.^(1,2)^

Although a large proportion of pregnant women infected with the coronavirus experience a smooth progression without complications, it is known that COVID-19 results in higher maternal, and fetal morbidity and mortality.^(3-5)^ In particular, there is concern regarding the impact of infection during pregnancy on perinatal complications.^(6-8)^ However, some studies have not found a clear association between COVID-19 and these complications.^(9,10)^ Nevertheless, paying special attention to this aspect is crucial due to the potential to alter child development.^(11)^

COVID-19 can lead to adverse outcomes such as abortion, premature birth, fetal growth restriction, and fetal death.^(4,12-20)^ However, there is still controversy regarding whether COVID-19 infection affects fetal growth, as some studies have not observed this association.^(7,19,21)^ Birth weight, as an indicator of immediate health conditions, can be correlated with neonatal morbidity and overall child development.^(22)^

Hence, COVID-19 infection has the potential to affect the health and development of an entire generation of children, with unpredictable consequences. This requires a healthcare system capable of addressing the challenges posed by this demand. The present study aims to evaluate the incidence and associated factors of small for gestational age (SGA) newborns in pregnant women infected with COVID-19.

## Methods

This study was a secondary analysis of data from the Brazilian REBRACO Network, a national multicenter cohort study that aimed to understand the impact of COVID-19 during pregnancy in a Brazilian obstetric population. Data for outpatient and inpatient data were collected from 01 February 2020 to 28 February 2021, before the implementation of vaccination against COVID-19 infection.

Eligibility criteria included pregnant women who attended obstetrical services of the participating centers presenting flu-like symptoms.

Flu-like symptoms considered were fever, cough, shortness of breath, sputum production, nasal or conjunctival congestion, difficulty swallowing, sore throat, runny nose, and clinical signs of respiratory distress or effort, such as O2 saturation <95%, signs of cyanosis, flapping of the nose, intercostal retraction, dyspnea, diarrhea, anosmia, and dysgeusia.

The COVID-19 was confirmed by laboratory (RT-PCR) and/or radiological pulmonary findings. Other symptomatic COVID-19 infection criteria included coughing, fever, nausea, vomiting, tachypnea, dyspnea, and chest pain. Participants (presenting with at least one of the symptoms mentioned above) were tested for SARS-CoV-2 infection according to local availability of testing and were submitted to laboratory exams and/or CT scans following local clinical protocols.

For the evaluation of pregnancy outcomes, only women who tested for COVID-19 and whose follow-up was considered successful (childbirth information and COVID-19 status available) were included in the analysis. Women with unavailable late pregnancy outcomes (unknown mode of delivery and gestational age at delivery), postpartum women at enrollment, and women with an ongoing pregnancy at the time of closure of the database were not considered.

We collect information on characteristics of sociodemographic, pregnancy, medical history, and the initial clinical presentation of COVID-19. After the clinical presentation of a case of COVID-19, the women were followed until childbirth and postpartum.

Study data were collected and managed using REDCap®15 (Research Electronic Data Capture) tools hosted at CAISM/UNICAMP server, the coordinating center. Research collaborators had hierarchical and clustered access to the system; data was properly anonymized, and personal contact information was kept confidential.

The definition of small for gestational age (SGA) is based on concepts with birth weight below the tenth percentile, as described by Melamed et al.^(14)^ and Gordijn et al.^(16)^ To determine SGA status, we used the GROW Birthweight Centile growth table developed by Gardosi et al.^(23)^

In our study, we assessed the potential risk factors for SGA based on those identified for the Brazilian population, including age, race, hypertensive diseases, pre-gestational diabetes, autoimmune diseases, antiphospholipid antibody syndrome, maternal malnutrition, and tobacco use, as reported by Souza et al.^(24)^ For our analysis, we considered infants who were classified as large for gestational age (LGA) as non-SGA.

For maternal weight definitions, we used the values recorded on the prenatal card. Maternal BMI was classified as follows: underweight (< 18.5 kg/m2), normal weight (18.5-24.9 kg/m2), overweight (25.0-29.9 kg/m2), and obesity (≥ 30.0 kg/m2).

We analyzed the incidence of small for gestational age (SGA) infants within our study population, differentiating between those who were infected with COVID-19 and those who were uninfected. We compared SGA newborns with non-SGA newborns, focusing on the following variables: risk of cesarean delivery, prematurity, Apgar score <7 at the 5th minute, admission to the Neonatal ICU, use of mechanical ventilation, and neonatal death.

We report the number of women with positive COVID-19 infection, the proportion of cases investigated (COVID-19 tests carried out), and the cases confirmed for all participants in the period considered.

We compared sociodemographic characteristics (age, education, marital status, pre-gestational body mass index - BMI and country region, pregnancy conditions (multiple pregnancies, parity, planned or unplanned pregnancy, and type of prenatal insurance), and medical condition characteristics (alcohol use, asthma, chronic kidney disease, diabetes, hypertension, and smoking) of women who attended public and private hospitals for childbirth. For descriptive purposes, the North and Northeast Brazilian regions were grouped. To assess obstetric outcomes, the following variables were evaluated: mode of birth, miscarriage, fetal death, prematurity (any childbirth <37 weeks), pre-eclampsia (new onset of hypertension, blood pressure higher or equal to 140×90mmHg in two or more measures, after 20 weeks of gestation with proteinuria or other laboratory or clinical signs of organ dysfunction), birth weight (adequacy of birth weight according to gestational age using the GROW customized chart), Apgar score (below 7 at 5 minutes), respiratory distress, admission to NICU (neonatal intensive care unit), and neonatal death.

Information on the severity of COVID-19 infection included severe acute respiratory syndrome (SARS), admission to the ICU, need for intubation and prone position, renal impairment, maternal death, and any severe maternal outcomes. Severe maternal outcomes (SMO) were defined as having any of the following: SARS, admission to ICU, or maternal death.

Women were divided into two groups, with SGA and non-SGA concepts. For comparisons using qualitative variables, Chi-squared or Fisher's Exact tests were used when appropriate to assess statistical significance between groups. For the analysis of quantitative variables, Pearson and Spearman correlations were used according to the distribution of normality. To determine the association of COVID-19 infection with pregnancy outcomes in SGA and non-SGA concepts, we estimated unadjusted risk ratios with their 95% confidence intervals.

We use SPSS 26.0 (IBM Corp. Released 2019. IBM SPSS Statistics for Windows, Version 26.0. Armonk, NY: IBM Corp) for statistical analysis.

**Figure 1:**
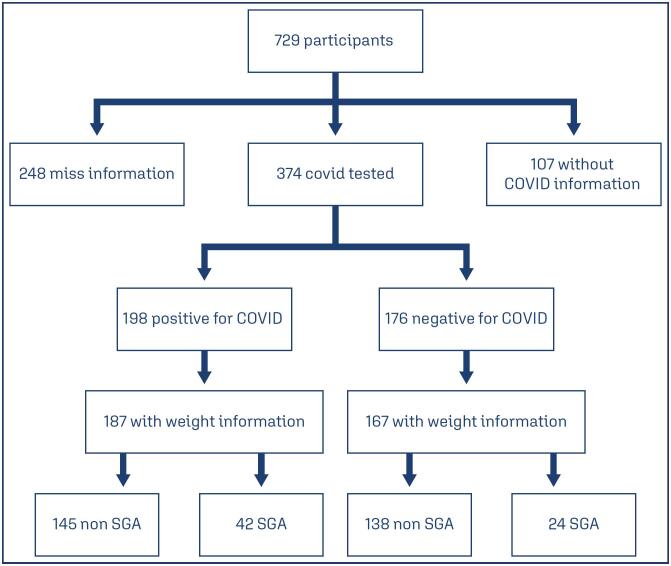
Study participants

The study protocol followed the Declaration of Helsinki amended in 1964 and it was approved by the Institutional Review Board (IRB) of the coordinating center and by each participating center (*Certificado de Apresentação de Apreciação Ética*: 31591720.5.3005.5330) protocol 5.464.119. The Strengthening the Reporting of Observational Studies in Epidemiology (STROBE) guidelines were followed for the implementation and reporting of the study. All included women received detailed information about the study and provided informed consent to their participation before enrollment (Figure 1).

## Results

This sample consisted of 729 initially symptomatic pregnant women with COVID-19. Among them, 248 participants who had not yet given birth at the time of data closure or had unavailable delivery information (due to missing data or loss of follow-up) and 107 who did not have confirmatory tests for COVID-19 were excluded from the analysis. As a result, a total of 374 women were included in the study. Out of the remaining participants, 198 tested positive for COVID-19, while 176 tested negative (Figure 1). Table 1 displays the distribution of certain demographic data among symptomatic pregnant women based on their testing results for COVID-19. Among pregnant women with confirmed COVID-19, 17% required ICU admission. 23,8% of SGA newborns were born to mothers who had severe infection. The incidence of small for gestational age (SGA) infants was found to be 22.4% among women who tested positive for COVID-19, while the incidence of SGA among women who tested negative for COVID-19 was 14.8%.

**Table 1:** Demographic data of symptomatic pregnant women tested for COVID-19

Variables		Testing for COVID-19		p-value
		Positive n(%)	Negative n(%)	
Age				
	≤19	9(4.5)	19(10.8)	0.037
	20-35	143(72.2)	127(72.2)	
	>35	46(23.2)	30(17.0)	
Ethnicity				
	White	112(57.4)	95(54.6)	0.583
	Non-white	83(42.6)	79(45.4)	
Marital status				
	With partner	131(67.2)	101(58.0)	0.070
	Without partner	64(32.8)	73(42.0)	
Country regions				
	North, Northeast	28(14.1)	6(3.4)	0.000
	Southeast	125(63.1)	149(84.7)	
	South	45(22.7)	21(11.9)	
Health Service				
	Public	154(77.8)	156(88.6)	0.005
	Private	44(22.2)	20(11.4)	
Education				
	Primary or lower	44(27.2)	67(42.1)	0.005
	Secondary or higher	118(72.8)	92(57.9)	
Body Mass Index (BMI)				
	Low weight(<18.50)	1(0.7)	4(3.8)	0.064
	Eutrophic (18.50-24.99)	41(28.9)	34(32.7)	
	Overweight (25.00-29.99)	50(35.2)	23(22.1)	
	Obese(≥30)	50(35.2)	43(41.3)	

Among pregnant women who had confirmed COVID-19, table 2 presents the demographic and clinical characteristics based on the condition of the newborn, whether they were classified as small for gestational age (SGA) or non-SGA.

**Table 2:** Demographic and clinical characteristics according to newborn weight adequacy among pregnant women with confirmed COVID-19

Variables		SGA n(%)	Non SGA n(%)	p-value
Age				
	≤19	2(4.8)	5(3.4)	0.909
	20-35	30(71.4)	107(73.8)	
	>35	10(23.8)	33(22.8)	
Ethnicity				
	White	21(50.0)	86(59.7)	0.262
	Non white	21(50.0)	58(40.3)	
Marital status				
	With partner	29(69.0)	95(66.9)	0.794
	Without partner	13(31.0)	47(33.1)	
Country regions				
	North/ Northeast	10(23.8)	16(11.0)	0.007
	Southeast	29(69.0)	89(61.4)	
	South	3(7.1)	40(27.6)	
Health service				
	Public	36(85.7)	109(75.2)	0.149
	Private	6(14.3)	36(24.8)	
Pre natal care				
	Public	30(81.1)	102(72.9)	0.307
	Private / Health insurance / Mixed	7(18.9)	38(27.1)	
Education				
	Primary or lower	13(37.1)	29(24.4)	0.136
	Secondary or higher	22(62.9)	90(75.6)	
Number of gestations				
	Nulliparous	14(33.3)	45(31.2)	0.799
	Multiparous	28(66.7)	99(68.8)	
Body Mass Index (BMI)				
	Low weight (<18.50)	1(3.7)	0(0)	0.163
	Eutriphic (18.50-24.99)	8(29.6)	29(26.9)	
	Overweight (25.00-29.99)	7(25.9)	41(38.0)	
Obese (≥30)		11(40.7)	38(35.2)	
Pregnancy trimester – manifestation of symptoms				
	1º trimester	7(16.7)	12(8.3)	0.281
	2º trimester	10(23.8)	36(24.8)	
	3º trimester	25(59.5)	97(66.9)	
Smoking				
	Yes	0(0)	1(0.7)	0.589
	No	42(100.0)	144(99.3)	
Asthma (pre-existing)				
	Yes	3(7.1)	10(6.9)	0.956
	No	39(92.9)	135(93.1)	
Chronic hypertension (pre-existing)				
	Yes	1(2.4)	17(11.7)	0.071
	No	41(97.6)	128(88.3)	
Pre-eclampsia				
	Yes	4(9.5)	17(12.0)	0.661
	No	38(90.5)	125(88.0)	
Presence of overweight/obesity				
	Yes	18(66.7)	79(73.1)	0.503
	No	9(33.3)	29(26.9)	
Gestacional diabetes				
	Yes	0(0)	2(1.4)	0.444
	No	42(100.0)	143(98.6)	
MEOWS				
	≥4	18(54.5)	49(45.0)	0.334
	<4	15(45.5)	60(55.0)	
Severe Acute Respiratory Syndrome (SARS)				
	Yes	8(19.0)	19(13.1)	0.334
	No	34(81.0)	126(86.9)	
ICU indication				
	Yes	10(23.8)	22(15.2)	0.191
	No	32(76.2)	123(84.8)	

Table 3 compares the birth outcomes between SGA and non-SGA newborns. SGA was not associated with an increase in the rate of cesarean delivery or low Apgar score at birth. However, it was associated with preterm birth, admission to the neonatal ICU, the use of mechanical ventilation, and neonatal death. It was observed that SGA newborns in symptomatic pregnant women have a 1.9 times higher likelihood of experiencing adverse outcomes (prematurity, fetal death, neonatal death, and admission to the neonatal ICU) compared to non-SGA newborns [odds ratio (OR) = 1.938, 95% confidence interval (CI): 1.472 - 2.552, P < 0.000]. When analyzing only pregnant women confirmed for COVID-19 (as shown in Table 3), we observe that the adequacy of the newborn's weight (SGA and non-SGA) is influenced primarily by prematurity. Specifically, prematurity is associated with higher rates of admission to the neonatal ICU and the use of mechanical ventilation. However, the risk of cesarean section, Apgar score lower than seven at the fifth minute of life, and the occurrence of neonatal death did not demonstrate a significant relationship in the studied sample.

**Table 3:** Neonatal outcomes among COVID-19 positive pregnant women

Variables	SGA (%)	Non SGA (%)	RR (IC 95%)*	p-value
Risk of cesarean delivery	71.4	64.1	1.114 (0.888-1.397)	0.461
Prematurity	50.0	24.8	2.014 (1.331-3.048)	0.004
Apgar <7 (5th minute)	7.7	4.2	1.846 (0.483- 7.050)	0.404
Admission to the neonatal ICU	47.4	22.1	2.139 (1.355-3.378)	0.004
Use of mechanical ventilation	30.8	9.5	3.243 (1.611-6.526)	0.003
Neonatal death	10.3	2.2	4.718 (1.102-20.197)	0.043

* RR – relative risk;

CI – confidence interval

It was observed that SGA newborns, in COVID-19 positive pregnant women, have a 1.6 times higher likelihood of experiencing adverse outcomes (prematurity, fetal death, neonatal death, and admission to the neonatal ICU) compared to non-SGA newborns [odds ratio (OR) = 1.655, 95% confidence interval (CI): 1.145 - 2.394, P = 0.017]. When comparing SGA newborns between those with positive COVID-19 infection (n=42) and those with negative COVID-19 infection (n=24), concerning various birth outcomes, it was observed that only the use of mechanical ventilation was associated with confirmed COVID-19 infection. However, all other outcomes assessed in table 4 did not show a significant association. It is important to note that the total sample size for this analysis was 66 women.

**Table 4:** Neonatal outcomes were compared between SGA newborns from COVID-positive patients and SGA newborns from COVID-negative patients

Variables	SGA (COVID +) (%)	SGA (COVID -) (%)	RR (IC 95%)*	p-value
Risk of cesarean delivery	71.4	79.2	0.902 (0.682-1.194)	0.569
Prematurity	50.0	33.3	1.500 (0.790-2.849)	0.210
Apgar <7 (5th minute)	7.7	4.5	1.692 (0.187-15.304)	1.000
Admission to the Neonatal ICU	47.4	47.8	0.990 (0.576-1.704)	1.000
Use of mechanical ventilation	30.8	0.0	0.692 (0.562-.853)	0.002
Neonatal death	10.3	0.0	0.897 (0.807-.998)	0.287

* RR – relative risk;

CI – confidence interval

It was observed that in the group of SGA newborns from COVID-19 positive pregnant women, there is a 1.07 times higher likelihood of experiencing adverse outcomes compared to the SGA newborns from COVID-19 negative pregnant women. However, this difference did not reach statistical significance, indicating that the observed association may have occurred by chance and is not statistically significant. When evaluating the trimester of pregnancy for COVID-19 infection, no statistically significant difference was identified in terms of outcomes. However, it is important to acknowledge that this lack of significance may be attributed to the sample size (represented by the variable "n") used in the analysis. A larger sample size may be necessary to detect potential differences and establish more conclusive results.

## Discussion

The incidence of SGA newborns in our study was 22.4%, which is higher compared to national data. This higher incidence of small-for-gestational-age fetuses may be attributed to flu-like syndrome and not exclusively to COVID-19. Among symptomatic pregnant women, irrespective of COVID-19 confirmation, it was observed that SGA fetuses were more likely to be born prematurely, require neonatal ICU admission, and need mechanical ventilation, which are expected outcomes in the presence of prematurity.^(25)^ SGA preterm infants have a higher rate of neonatal morbidity and mortality compared to premature AGA infants. An important question to consider is whether these infants were SGA as a result of COVID-19 infection or due to premature births, failing to achieve their appropriate growth potential.

When pregnant women with confirmed COVID-19 were analyzed, prematurity was once again associated with weight gain, as well as neonatal ICU admission and the use of mechanical ventilation. Similarly, SGA newborns from pregnant women with confirmed COVID-19 had a higher risk of adverse outcomes.^(20)^ Both groups did not differ significantly in terms of cesarean section rates. These findings support studies that have evaluated fetuses from mothers symptomatic of COVID-19, demonstrating an increased risk of adverse effects.^(20)^ When comparing symptomatic pregnant women with confirmed COVID-19 and those negative for COVID-19, it was observed that SGA newborns born to infected pregnant women had a higher risk of requiring mechanical ventilation but no increased risk of prematurity, as reported by Di Mascio et al.^(20)^ More broadly, it should be noted that the impact of COVID-19 on obstetric and neonatal outcomes is low, and there seems to be no greater risk of fetal growth restriction or an increased incidence of small for gestational age fetuses in the studied population. These findings align with previous studies that have reported a low impact of COVID-19 on obstetric and neonatal outcomes.^(26)^ However, it is important to mention that the present study did not assess the severity of infection during pregnancy, which appears to be strongly associated with worse obstetric and neonatal outcomes.^(27)^ Additionally, our study did not evaluate neonatal infection, but there is evidence suggesting potential transplacental transmission,^(28,29)^ which speculates a greater need for mechanical ventilation in newborns from confirmed COVID-19 pregnancies due to the presence of respiratory complications associated with COVID-19 that amplify the typical stress observed during the neonatal period.^(27)^

On the other hand, our findings did not demonstrate higher cesarean section rates, consistent with previous data.^(20,27,30)^ The medical indication for cesarean section, in many studies, has been shown to be a consequence of maternal complications of COVID-19, including pneumonia.^(30)^ It is important to note that the lack of prior knowledge about COVID-19 infection and its maternal and fetal effects may have contributed to an iatrogenic increase in the rates of prematurity and cesarean section.

Most cases of growth restriction are attributed to placental dysfunction.^(31)^ The COVID-19 virus has an affinity for angiotensin-converting enzyme 2 receptors, which are widely expressed in the placenta.^(28,29)^ Therefore, it is presumed that placental dysfunction could occur due to the presence of the virus, leading to obstetric complications such as abortion, growth restriction, and even preterm labor.^(30)^ However, the current literature does not provide evidence of an increased incidence of fetal growth restriction in COVID-19-positive patients.^(26)^

Birth weight is an important factor that determines perinatal morbidity and mortality, and it can also affect metabolic, respiratory, and immune development. Therefore, it is crucial to evaluate birth weight in all pregnancies, particularly in pregnant women with comorbidities, including COVID-19.^(25)^

COVID-19 has been associated with greater maternal and neonatal adverse outcomes compared to pregnant women without the infection.^(32)^ Although a slight impact has been observed in infected pregnant women^(7)^, this population should be considered high-risk and closely monitored during prenatal care. A study conducted in low-income countries may have results that can be extrapolated to the Brazilian context due to financial disparities. Burt et al.^(33)^ evaluated the indirect effects of public policies related to the pandemic and restricted access to newborn and postnatal care. Therefore, neonatal and pediatric outcomes may be worse than initially assessed.

Consistently, COVID-19 infection has been significantly associated with prematurity,^(18)^ which is a major factor contributing to increased neonatal morbidity and mortality. Even late preterm infants have significantly higher rates of morbidity, mortality, and long-term neuropsychomotor development impairment compared to full-term infants.^(34)^ Iatrogenic preterm delivery accounts for approximately 25% of preterm births.^(35)^ Therefore, a significant portion of the increased risks of prematurity and SGA newborns may be attributed to iatrogenesis resulting from the lack of medical knowledge regarding a disease that reached alarming rates. Considering this, prenatal care for these pregnant women, along with maternal and fetal surveillance, general vaccination guidance, and pregnancy planning, are important measures to reduce risks.

An international study examined the outcomes of pregnant women who tested positive for COVID-19 or SARS-CoV-2. It evaluated 388 pregnancies across, most cases were diagnosed in the third trimester, with common symptoms like cough and fever. It found that 11.1% required ICU admission, with a maternal mortality rate of 0.8%. Adverse fetal outcomes, including perinatal death, were observed in 4.2% of pregnancies completed to term, but no congenital anomalies were noted in stillbirths or neonatal deaths. Factors like earlier diagnosis and maternal respiratory support were associated with worse outcomes, while treatments like hydroxychloroquine showed no significant effect. The study also indicated a minimal risk of vertical transmission but emphasized the need for further research.^(36)^

A recent study investigated pregnant women infected with SARS-CoV-2 during their first or second trimesters to identify potential effects on fetal central nervous system (CNS) development. The study used neurosonography to assess CNS development markers and found no differences compared to a control group.^(37)^ Similarly, another study focused on potential cardiovascular changes and blood flow in the umbilical vein of women infected in the second trimester, determining that mild SARS-CoV-2 infection does not increase the risk of fetal hemodynamic or cardiac issues.^(38)^ These findings suggest that SARS-CoV-2 infection does not affect fetal cortical or cardiovascular development, indicating that additional testing during pregnancy to rule out these conditions is not necessary.

A study evaluated the anxiety levels of pregnant women during the COVID-19 pandemic. Media, particularly newspapers, TV, and the Internet, were primary sources of COVID-19 information. Anxiety levels were high, notably concerning pregnancy outcomes, and correlated with higher education levels. The study underscores the pandemic's significant impact on maternal anxiety, highlighting the importance of tailored support and reliable information dissemination for pregnant women during health crises.^(39)^

## Conclusion

These studies illustrate the significant impact that infection can have on the physical and mental health of pregnant women. Understanding these effects is essential for offering appropriate support and care during these difficult times. These findings underscore the need for ongoing research and personalized management strategies to safeguard maternal and fetal health. All evidence-based information about fetal development, especially important systems such as the CNS and cardiovascular, needs to be communicated to pregnant women for reassuring about the health of their fetuses and the low risk of adverse outcomes. It is important to acknowledge that our study had certain limitations, including the focus on fetal growth evaluation rather than investigating infection in newborns. By analyzing these neonatal outcomes in SGA newborns from COVID-positive and COVID-negative patients, the study aimed to provide insights into the potential effects of COVID-19 on the health and well-being of SGA infants. Further studies are needed to assess the long-term outcomes of these SGA newborns born to COVID-19-infected patients.
